# Supplementary material for: Predicting recombination frequency from map distance
Source: Heredity (Edinb). 2022 Dec 24;130(3):114–21. doi: 10.1038/s41437-022-00585-3 (PMC9981558; doi:10.1038/s41437-022-00585-3)
Supplement: Supplementary file 1 — Supplementary material [file 41437_2022_585_MOESM1_ESM.pdf]

Supplementary material for: Predicting recombination frequency from map distance

Mikko Kivikoski[a,1], Pasi Rastas[b], Ari Löytynoja[b], Juha Merilä[a,c]

[a] Ecological Genetics Research Unit, Organismal and Evolutionary Biology Research Programme, Faculty of Biological and Environmental Sciences, FI-00014 University of Helsinki, Finland

[b] Institute of Biotechnology, HiLIFE, FI-00014 University of Helsinki, Finland

[c] Area of Ecology and Biodiversity, School of Biological Sciences, Kadoorie Biological Science Building, Pokfulam Road, The University of Hong Kong, Hong Kong SAR

[1] corresponding author: Mikko Kivikoski, E-mail: mikko.kivikoski@helsinki.fi

TABLE OF CONTENTS

SUPPLEMENTARY METHODS 3

Using gametic crossovers to interpret crossover localization in the bivalent 3

Proofs for three markers 4

REFERENCES 6

SUPPLEMENTARY TABLES 7

Tables S1–S6 7

Table S7. 8

Table S8. 9

Table S9. 10

SUPPLEMENTARY FIGURES 11

Figure S1. 11

Figure S2. 12

Figure S3. 13

Figure S4. 14

Figure S6. 16

Figure S7. 17

Figure S8. 18

Figure S9. 19

Figure S10. 20

Figure S11. 21

Figure S12. 22

Figure S13. 23

Supplementary methods

Using gametic crossovers to interpret crossover localization in the bivalent

Linkage maps can be used to infer crossovers and their localization in gametes. However, gametic crossovers are a subset of those in the bivalent and the number of bivalent crossovers in the meiosis that preceded the gamete is generally unknown. This limits the possibilities to study the relationship between crossover count and localization from gametic data (Veller et al. 2022). Despite these limitations, the likelihood for bivalent crossover count preceding the gamete can be calculated. Assuming no chromatid interference, the likelihood for a gamete with *k* crossovers being from a bivalent with *k* crossovers is:

$$P(CO_{bivalent} = k | CO_{gamete} = k) = \frac{P(CO_{gamete} = k | CO_{bivalent} = k)}{P(CO_{gamete} = k)} = \frac{\frac{\binom{k}{k}}{2^k} p_k}{\sum_{l=k}^{n} \frac{\binom{l}{k}}{2^l} p_l} = \frac{\frac{1}{2^k} p_k}{\sum_{l=k}^{n} \frac{\binom{l}{k}}{2^l} p_l} \quad (1)$$

In Eq. 1 n is the maximum number of bivalent crossovers, p_k and p_l are the likelihoods for k and l bivalent crossovers, respectively. For example, in chromosome 8 (LG8) of the nine-spined stickleback, the inferred probabilities for 2-4 maternal crossovers are 0.85, 0.12 and 0.03, respectively, and probabilities for other crossover counts are 0 (Table S1). The likelihood for a gamete with two maternal crossovers representing the total number of crossovers in meiosis is therefore:

$$\frac{\frac{1}{2^2} p_2}{\sum_{l=2}^{4} \frac{\binom{l}{2}}{2^l} p_l} = \frac{\frac{1}{4} 0.85}{\frac{1}{4} 0.85 + \frac{3}{8} 0.12 + \frac{6}{16} 0.03} = 0.79 \quad (2)$$

Tables S1–S6 list the inferred likelihoods for different bivalent crossover counts and for the mode the likelihood that a gamete with the same number of crossovers is equal to the bivalent crossover count.

Proofs for three markers

According to Karlin and Liberman (1978), recombination frequencies r_{ij}, r_{jk} and r_{ik} between three markers *i, j* and *k* at map positions m_i, m_j, and m_k ($m_i \leq m_j \leq m_k$) should meet the criterion $r_{ij} + r_{jk} \geq r_{ik}$. Here, we show that this holds for the recombination frequencies predicted with the $p_0(k)$ function formulated in this paper. The three cases are (1) all three markers occur in the same crossover region, (2) two markers (here m_i and m_j) are in the same region and the third marker is in the next region and (3) all three markers are in three adjacent regions. If there is a marker-free region either between m_i and m_j or m_j and m_k, equation $r_{ij} + r_{jk} \geq r_{ik}$ holds, since $r_{ik} = 1/2$ and $r_{ij} + r_{jk} \geq 1/2$ as either r_{ij} or r_{jk} is 1/2 and the other one must be ≥ 0.

Case 1: m_i, m_j, and m_k are in the same crossover region, i.e. $\left\lceil \frac{m_k}{\frac{d}{k}} \right\rceil = \left\lceil \frac{m_j}{\frac{d}{k}} \right\rceil = \left\lceil \frac{m_i}{\frac{d}{k}} \right\rceil$

$$\begin{aligned} r_{ij} + r_{jk} &\geq r_{ik} \Leftrightarrow \\ \frac{1}{2}\left(1 - p_0(k)_{m_i,m_j}\right) + \frac{1}{2}\left(1 - p_0(k)_{m_j,m_k}\right) &\geq \frac{1}{2}\left(1 - p_0(k)_{m_i,m_k}\right) \Leftrightarrow \\ \frac{1}{2}\left(1 - \left(1 - \frac{|m_j - m_i|}{\frac{d}{k}}\right)\right) + \frac{1}{2}\left(1 - \left(1 - \frac{|m_k - m_j|}{\frac{d}{k}}\right)\right) &\geq \frac{1}{2}\left(1 - \left(1 - \frac{|m_k - m_i|}{\frac{d}{k}}\right)\right) \Leftrightarrow \\ \frac{|m_j - m_i|}{\frac{d}{k}} + \frac{|m_k - m_j|}{\frac{d}{k}} &\geq \frac{|m_k - m_i|}{\frac{d}{k}} \Leftrightarrow \\ |m_j - m_i| + |m_k - m_j| &\geq |m_k - m_i| \Leftrightarrow \\ |m_k - m_i| &\geq |m_k - m_i| \end{aligned} \tag{3}$$

Case 2: $\left\lceil\frac{m_k}{\frac{d}{k}}\right\rceil$ - $\left\lceil\frac{m_j}{\frac{d}{k}}\right\rceil = 1$; $\left\lceil\frac{m_j}{\frac{d}{k}}\right\rceil = \left\lceil\frac{m_i}{\frac{d}{k}}\right\rceil$; and $b_j = b_i$

$$
\begin{aligned}
r_{ij} + r_{jk} \geq r_{ik} \Leftrightarrow \\
\frac{1}{2}\left(1 - p_0(k)_{m_i,m_j}\right) + \frac{1}{2}\left(1 - p_0(k)_{m_j,m_k}\right) \geq \frac{1}{2}\left(1 - p_0(k)_{m_i,m_k}\right) \Leftrightarrow \\
\frac{1}{2}\left[1 - \left(1 - \frac{|m_j - m_i|}{\frac{d}{k}}\right)\right] + \frac{1}{2}\left[1 - \left(1 - \frac{|b_j - m_j|}{\frac{d}{k}}\right) \cdot \frac{|b_k - m_k|}{\frac{d}{k}}\right] \geq \frac{1}{2}\left[1 - \left(1 - \frac{|b_i - m_i|}{\frac{d}{k}}\right) \cdot \frac{|b_k - m_k|}{\frac{d}{k}}\right] \Leftrightarrow ||b_j = b_i \\
1 - 1 + \frac{|m_j - m_i|}{\frac{d}{k}} + 1 - \frac{|b_k - m_k|}{\frac{d}{k}} + \left(\frac{|b_j - m_j|}{\frac{d}{k}} \cdot \frac{|b_k - m_k|}{\frac{d}{k}}\right) \geq 1 - \frac{b_k - m_k}{\frac{d}{k}} + \left(\frac{|b_j - m_i|}{\frac{d}{k}} \cdot \frac{|b_k - m_k|}{\frac{d}{k}}\right) \Leftrightarrow \\
\frac{|m_j - m_i|}{\frac{d}{k}} - \frac{|b_k - m_k|}{\frac{d}{k}} + \left(\frac{|b_j - m_j|}{\frac{d}{k}} \cdot \frac{|b_k - m_k|}{\frac{d}{k}}\right) \geq - \frac{b_k - m_k}{\frac{d}{k}} + \left(\frac{|b_j - m_i|}{\frac{d}{k}} \cdot \frac{|b_k - m_k|}{\frac{d}{k}}\right) \Leftrightarrow \\
\frac{|m_j - m_i|}{\frac{d}{k}} + \left(\frac{|b_j - m_j|}{\frac{d}{k}} \cdot \frac{|b_k - m_k|}{\frac{d}{k}}\right) \geq \left(\frac{|b_j - m_i|}{\frac{d}{k}} \cdot \frac{|b_k - m_k|}{\frac{d}{k}}\right) \Leftrightarrow \\
\frac{|m_j - m_i|}{\frac{d}{k}} \geq \frac{|b_j - m_i|}{\frac{d}{k}} \cdot \frac{|b_k - m_k|}{\frac{d}{k}} - \frac{|b_j - m_j|}{\frac{d}{k}} \cdot \frac{|b_k - m_k|}{\frac{d}{k}} \Leftrightarrow \\
\frac{|m_j - m_i|}{\frac{d}{k}} \geq \left(\frac{|b_j - m_i|}{\frac{d}{k}} - \frac{|b_j - m_j|}{\frac{d}{k}}\right) \cdot \frac{|b_k - m_k|}{\frac{d}{k}} \Leftrightarrow \\
\frac{|m_j - m_i|}{\frac{d}{k}} \geq \frac{|m_j - m_i|}{\frac{d}{k}} \cdot \frac{|b_k - m_k|}{\frac{d}{k}} \Leftrightarrow \\
1 \geq \frac{|b_k - m_k|}{\frac{d}{k}}
\end{aligned}
\tag{4}
$$

Case 3: $\left\lceil \frac{m_k}{\frac{d}{k}} \right\rceil - \left\lceil \frac{m_j}{\frac{d}{k}} \right\rceil = 1; \left\lceil \frac{m_j}{\frac{d}{k}} \right\rceil - \left\lceil \frac{m_i}{\frac{d}{k}} \right\rceil = 1$

$$
\begin{aligned}
r_{ij} + r_{jk} \geq r_{ik} \Leftrightarrow \\
\frac{1}{2}\left(1 - p_0(k)_{m_i,m_j}\right) + \frac{1}{2}\left(1 - p_0(k)_{m_j,m_k}\right) \geq \frac{1}{2}\left(1 - p_0(k)_{m_i,m_k}\right) \Leftrightarrow \\
\frac{1}{2}\left[1 - \left(1 - \frac{|b_i - m_i|}{\frac{d}{k}}\right) \cdot \frac{|b_j - m_j|}{\frac{d}{k}}\right] + \frac{1}{2}\left[1 - \left(1 - \frac{|b_j - m_j|}{\frac{d}{k}}\right) \cdot \frac{|b_k - m_k|}{\frac{d}{k}}\right] \geq \frac{1}{2}(1 - 0) \Leftrightarrow \\
1 - \left(1 - \frac{|b_i - m_i|}{\frac{d}{k}}\right) \cdot \frac{|b_j - m_j|}{\frac{d}{k}} + 1 - \left(1 - \frac{|b_j - m_j|}{\frac{d}{k}}\right) \cdot \frac{|b_k - m_k|}{\frac{d}{k}} \geq 1 \Leftrightarrow \\
(-1) \cdot \left(1 - \frac{|b_i - m_i|}{\frac{d}{k}}\right) \cdot \frac{|b_j - m_j|}{\frac{d}{k}} + (-1) \cdot \left(1 - \frac{|b_j - m_j|}{\frac{d}{k}}\right) \cdot \frac{|b_k - m_k|}{\frac{d}{k}} \geq -1 \Leftrightarrow \\
\left(1 - \frac{|b_i - m_i|}{\frac{d}{k}}\right) \cdot \frac{|b_j - m_j|}{\frac{d}{k}} + \left(1 - \frac{|b_j - m_j|}{\frac{d}{k}}\right) \cdot \frac{|b_k - m_k|}{\frac{d}{k}} \leq 1
\end{aligned}
\tag{5}
$$

The maximum value for the terms $\left(1 - \frac{|b_i - m_i|}{\frac{d}{k}}\right)$ and $\frac{|b_k - m_k|}{\frac{d}{k}}$ is 1.

Therefore, it is sufficient to investigate the equation by setting those values to 1:

$$
\begin{aligned}
1 \cdot \frac{|b_j - m_j|}{\frac{d}{k}} + \left(1 - \frac{|b_j - m_j|}{\frac{d}{k}}\right) \cdot 1 \leq 1 \Leftrightarrow \\
\frac{|b_j - m_j|}{\frac{d}{k}} + 1 - \frac{|b_j - m_j|}{\frac{d}{k}} \leq 1 \Leftrightarrow \\
1 \leq 1
\end{aligned}
$$

References

Karlin, S., & Liberman, U. (1978). Classifications and comparisons of multilocus recombination distributions. *Proceedings of the National Academy of Sciences USA*, 75: 6332–6336.

Veller, C., Wang, S., Zickler, D., Zhang, L., & Kleckner, N. (2022). Limitations of gamete sequencing for crossover analysis. *Nature*, 606: E1–E3.

Yu, K., & Feingold, E. (2001). Estimating the frequency distribution of crossovers during meiosis from recombination data. *Biometrics*, 57: 427–434.

Supplementary tables

Tables S1–S6 (separate csv files).

The number gametes with certain number of crossovers, total map lengths of the chromsosomes and the maximum-likelihood estimates for bivalent crossover counts estimated with the expected-maximization algorithm (Yu and Feingold 2001; see Methods in the main text).

Table names

1. Nine-spined stickleback female
2. Nine-spined stickleback male
3. Three-spined stickleback female
4. Three-spined stickleback male
5. Human female
6. Human male

Column names in the tables

- n[*]: number of gametes with [*] crossovers
- MLEp[*]: Maximum-likelihood estimate for probability of [*] crossovers in the bivalent.
- MLEpRestricted[*]: Maximum-likelihood estimate for probability of [*] crossovers in the bivalent, by restricting the likelihood for absence of crossovers to 0.
- BootstrapPvalue: Bootstrap *p*-value for the MLEp0. Low *p*-value indicates that non-zero MLEp0 is true finding and meiosis without crossovers may occur.
- MapLength: Map length (cM) of the chromosome.
- MapLengthParm: Map length (cM) of the shorter chromosome arm.
- MapLengthQarm: Map length (cM) of the longer chromosome arm.
- ModeBivalent: Inferred mode of number of crossovers in the bivalent (see columns MLEpRestricted[*]).
- PgameteMode: Probability that gamete with the 'ModeBivalent' crossover count descends from meiosis with the same number of crossovers (see Eq. 1 in Supplementary methods).

Table S7. Mean absolute error of the predicted recombination frequencies in the nine-spined stickleback. Lowest absolute values per sex and chromosome are in bold type.

	Maternal				Paternal			
Chromosome	Haldane	Kosambi	Linear	$p_0(k)$	Haldane	Kosambi	Linear	$p_0(k)$
LG1	0.0466	0.015	0.0193	**0.0095**	0.0083	0.0039	**0.0023**	0.0032
LG2	0.0478	0.0166	0.0145	**0.0103**	0.0084	0.0034	**0.0004**	0.0005
LG3	0.0378	0.0127	0.0155	**0.0096**	0.0091	0.004	0.0068	**0.0028**
LG4	0.0502	0.0183	0.0169	**0.0115**	0.0069	0.0031	0.0055	**0.0030**
LG5	0.0342	0.0115	0.013	**0.0023**	0.0142	0.006	**0.0006**	0.0046
LG6	0.0439	0.0168	0.0112	**0.0086**	0.0112	0.0048	**0.0007**	0.0041
LG7	0.0406	0.0148	0.012	**0.0079**	0.0111	0.0047	**0.0008**	0.0013
LG8	0.0456	0.0188	0.0134	**0.0088**	0.0079	0.0030	0.0040	**0.0018**
LG9	0.0405	0.0163	0.0127	**0.0065**	0.0139	0.0055	**0.0021**	0.0042
LG10	0.0378	0.0137	0.0139	**0.0085**	0.0124	0.0051	**0.0025**	0.0052
LG11	0.0414	0.013	0.0156	**0.0097**	0.0108	0.0048	**0.0017**	0.0028
LG12	0.043	0.0167	0.0216	**0.0142**	0.0094	0.0042	**0.0021**	0.0028
LG13	0.0387	0.0149	0.0175	**0.0102**	0.0098	0.0043	0.0062	**0.0040**
LG14	0.0394	0.0162	**0.0074**	0.0119	0.0152	0.0068	**0.0008**	0.0047
LG15	0.0363	0.0117	0.0168	**0.0085**	0.0127	0.0056	**0.0012**	0.0045
LG16	0.0424	0.0152	0.0155	**0.0085**	0.0106	0.0043	0.0017	**0.0013**
LG17	0.0376	0.0139	0.0193	**0.0085**	0.0094	0.0043	0.0040	**0.0029**
LG18	0.0346	0.0127	0.0095	**0.0072**	0.0158	0.0072	**0.0003**	0.0058
LG19	0.0384	0.0136	0.0154	**0.00400**	0.0098	0.0033	0.0039	**0.0011**
LG20	0.0378	0.015	0.0155	**0.0045**	0.0097	0.0033	0.0025	**0.0012**
LG21	0.0365	0.0136	0.0110	**0.0108**	0.0126	0.0053	**0.0011**	0.0026

Table S8. Mean absolute error of the predicted recombination frequencies in the three-spined stickleback. Lowest absolute values per sex and chromosome are in bold type.

	Maternal				Paternal			
Chromosome	Haldane	Kosambi	Linear	$p_0(k)$	Haldane	Kosambi	Linear	$p_0(k)$
chrI	0.0479	0.0161	0.015	**0.0087**	0.0081	0.0033	0.0061	**0.0021**
chrII	0.0541	0.0237	**0.0107**	0.0128	0.0086	0.0036	**0.0005**	0.0008
chrIII	0.0394	0.0146	0.0103	**0.0075**	0.0111	0.0042	0.0023	**0.0015**
chrIV	0.0533	0.0207	0.0165	**0.0108**	0.0060	0.0034	0.0063	**0.0025**
chrV	0.0358	0.0131	0.0094	**0.0088**	0.0095	0.0037	**0.0002**	0.0024
chrVI	0.0373	0.0127	0.0125	**0.0084**	0.0086	0.0034	**0.0004**	0.0015
chrVII	0.0507	0.0209	0.0175	**0.0154**	0.0077	0.0058	0.0108	**0.005**
chrVIII	0.0413	0.0141	0.0156	**0.0067**	0.0092	0.0041	**0.0012**	0.0017
chrIX	0.0406	0.0142	0.0145	**0.0071**	0.0109	0.0047	**0.0002**	0.0024
chrX	0.0375	0.0133	0.0113	**0.008**	0.010	0.0038	0.0009	**0.0007**
chrXI	0.0443	0.0189	**0.0071**	0.0159	0.0082	0.0032	**0.0007**	0.0014
chrXII	0.0393	0.0121	0.0168	**0.0046**	0.0073	0.0029	**0.0006**	0.0014
chrXIII	0.0444	0.0162	0.0126	**0.008**	0.0085	0.0035	**0.0007**	0.001
chrXIV	0.036	0.0154	**0.0055**	0.013	0.0111	0.0044	**0.0008**	0.0011
chrXV	0.0479	0.0204	**0.0078**	0.0127	0.0055	0.0018	0.0006	**0.0003**
chrXVI	0.0465	0.0203	**0.0119**	0.013	0.0106	0.005	**0.0025**	0.0047
chrXVII	0.0371	0.0125	0.0165	**0.0042**	0.0103	0.004	**0.0012**	0.0014
chrXVIII	0.0364	0.0125	0.0113	**0.0058**	0.0105	0.0042	0.0012	**0.0010**
chrXIX	0.0473	0.019	0.0181	**0.0107**	0.0102	0.0069	0.00675	**0.00673**
chrXX	0.0382	0.0154	0.022	**0.0066**	0.0067	0.0036	0.0041	**0.0030**
chrXXI	0.0307	**0.01040**	0.0185	0.01041	0.0102	0.0043	**0.0005**	0.0046

Table S9. Mean absolute error of the predicted recombination frequencies in human. Lowest absolute values per sex and chromosome are in bold type.

	Maternal				Paternal			
Chromosome	Haldane	Kosambi	Linear	$p_0(k)$	Haldane	Kosambi	Linear	$p_0(k)$
chr1	0.021	0.0056	0.007	**0.003**	0.0317	0.0108	0.0072	**0.0044**
chr2	0.0213	0.0051	0.0081	**0.0028**	0.0322	0.0111	0.0072	**0.004**
chr3	0.024	0.0062	0.0082	**0.0036**	0.0317	0.0108	0.0087	**0.0044**
chr4	0.024	0.006	0.0085	**0.0028**	0.0305	0.0103	0.0092	**0.0039**
chr5	0.0257	0.0068	0.008	**0.0029**	0.0307	0.0107	0.0089	**0.0037**
chr6	0.0264	0.0071	0.0082	**0.0034**	0.0282	0.0092	0.0096	**0.0045**
chr7	0.0265	0.007	0.0087	**0.0035**	0.0289	0.0093	0.0099	**0.0043**
chr8	0.0269	0.0073	0.0088	**0.0037**	0.0249	0.0074	0.0101	**0.003**
chr9	0.0276	0.0076	0.0088	**0.0038**	0.0251	0.0079	0.0112	**0.0056**
chr10	0.028	0.0081	0.0078	**0.0033**	0.0265	0.0084	0.0097	**0.0036**
chr11	0.0285	0.0081	0.0087	**0.0043**	0.0243	0.0071	0.0099	**0.0033**
chr12	0.0286	0.0082	0.0084	**0.0034**	0.0269	0.0088	0.0092	**0.0033**
chr13	0.031	0.0098	0.0092	**0.0043**	0.0253	0.0091	0.0071	**0.0026**
chr14	0.0298	0.009	0.0103	**0.0046**	0.0262	0.0098	0.0065	**0.0036**
chr15	0.03	0.009	0.0098	**0.005**	0.0223	0.0068	0.0084	**0.0021**
chr16	0.0297	0.0093	0.0076	**0.0042**	0.0236	0.0081	0.0081	**0.0014**
chr17	0.0308	0.0099	0.0078	**0.0036**	0.0212	0.0061	0.0088	**0.0016**
chr18	0.0318	0.0106	0.0084	**0.0043**	0.0204	0.0065	0.0062	**0.003**
chr19	0.0321	0.0114	0.0078	**0.0051**	0.0181	0.0059	0.007	**0.0021**
chr20	0.031	0.0103	0.0086	**0.0037**	0.0197	0.0065	0.0065	**0.0013**
chr21	0.022	0.0072	0.0074	**0.0049**	0.0157	0.0062	0.0007	**0.0006**
chr22	0.0239	0.0078	0.0086	**0.0054**	0.0173	0.0064	**0.0024**	0.0027

Supplementary figures

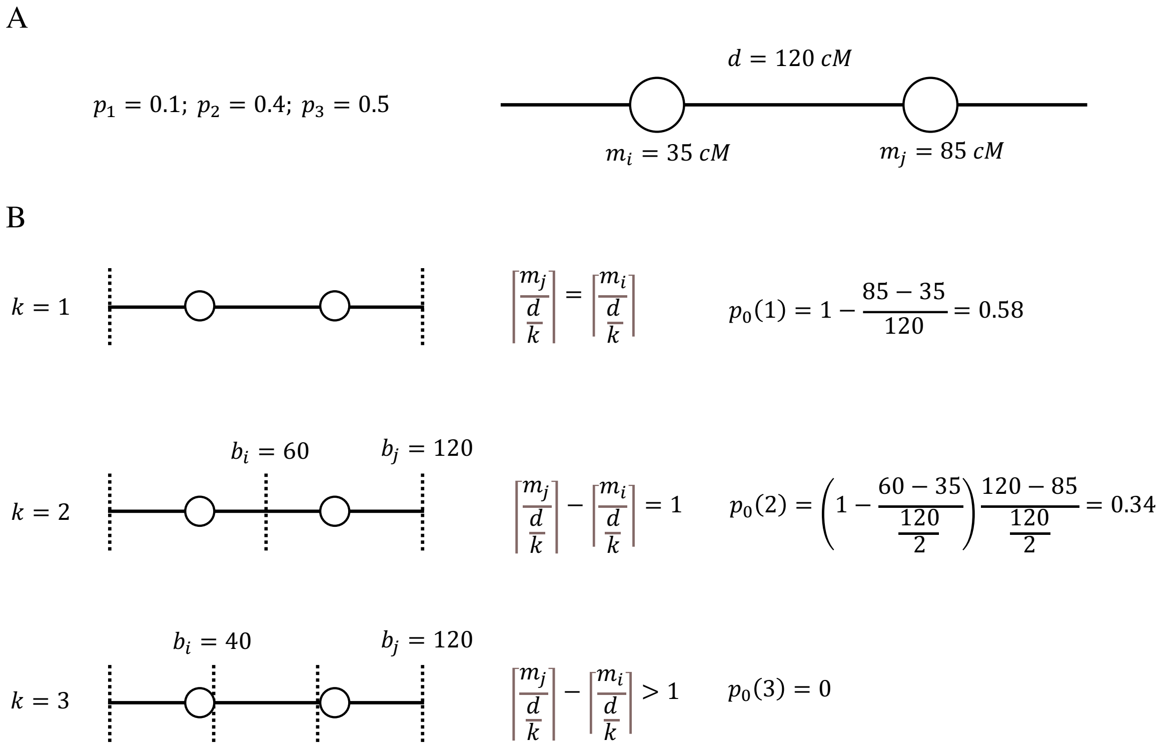

C

$$r = \frac{1}{2}(1 - p_0) = \frac{1}{2}\left(1 - \sum_{k=1}^{3} p_0(k) p_k\right) = \frac{1}{2}\big(1 - (0.1 \cdot 0.58 + 0.4 \cdot 0.34 + 0.5 \cdot 0)\big) = \frac{1}{2}(1 - 0.194) = 0.40$$

D

Inverse Haldane's mapping function: $r = \frac{1}{2}(1 - e^{-2 \cdot 0.5}) \approx 0.32$

Inverse Kosambi's mapping function: $r = \frac{1}{2}\big(\tanh(2 \cdot 0.5)\big) \approx 0.38$

Inverse linear: $r = 0.5$

Figure S1. Application of the $p_0(k)$ function. (A) Suppose a chromosome with a total map length (d) of 120 cM and the likelihoods of 0.1, 0.4 and 0.5 for 1, 2 or 3 bivalent crossovers, respectively (p_1, p_2, p_3), obtained from empirical data. (B) Probability for the absence of crossovers between the markers m_i and m_j is calculated with $p_0(k)$ function (Eq. 1 in the main text). Chromosome segmentation is indicated with dashed lines. (C) Recombination frequency, r, predicted assuming no chromatid interference and p_0 according to $p_0(k)$ function. (D) Recombination frequency predicted with inverse mapping functions.

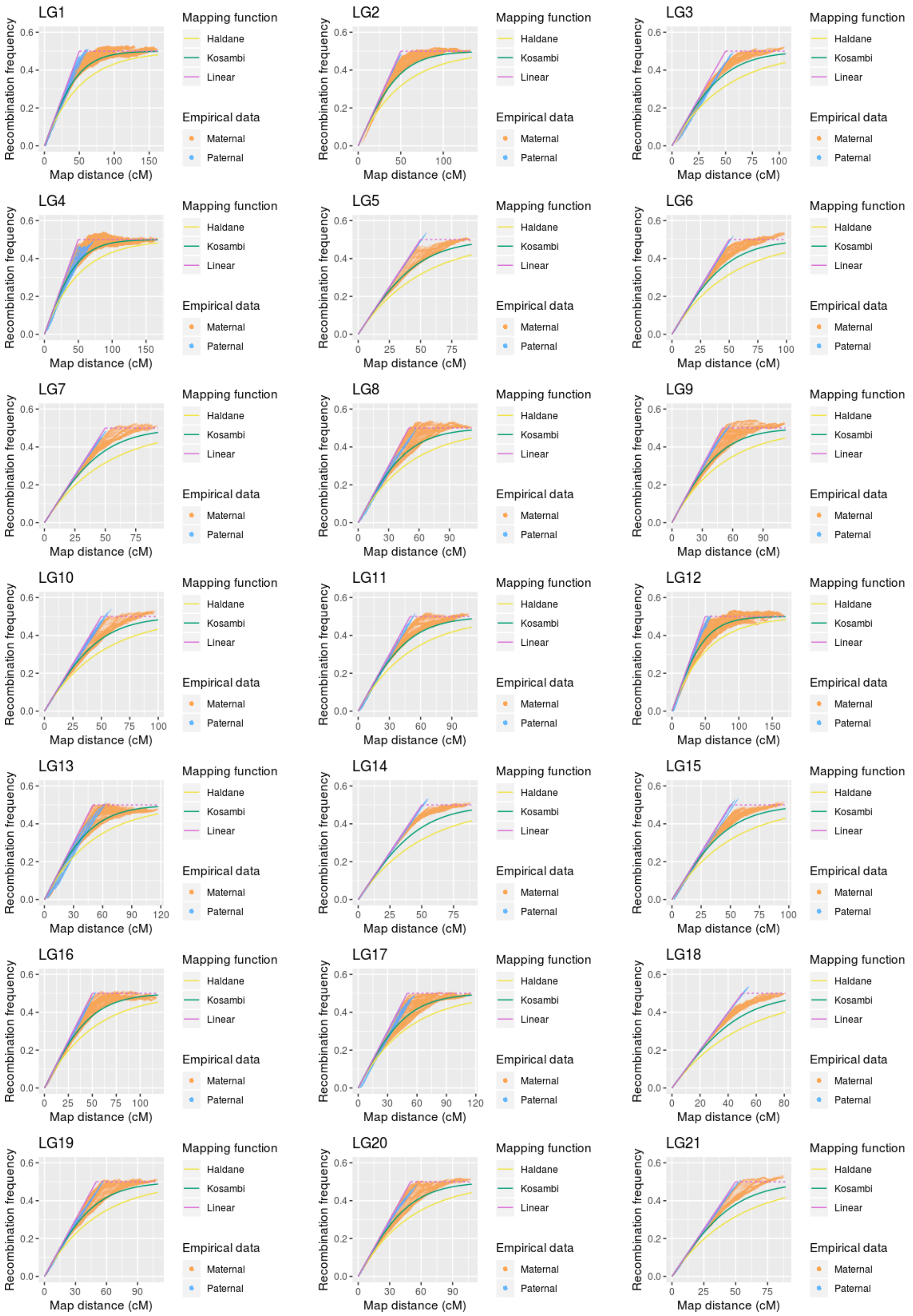

Figure S2. Empirical recombination frequencies in the 21 nine-spined stickleback chromosomes. Solid lines show the three inverse mapping functions; the linear function was capped at 0.5, shown with the dashed line. Each orange and blue dot is a marker pair in maternal and paternal data, respectively.

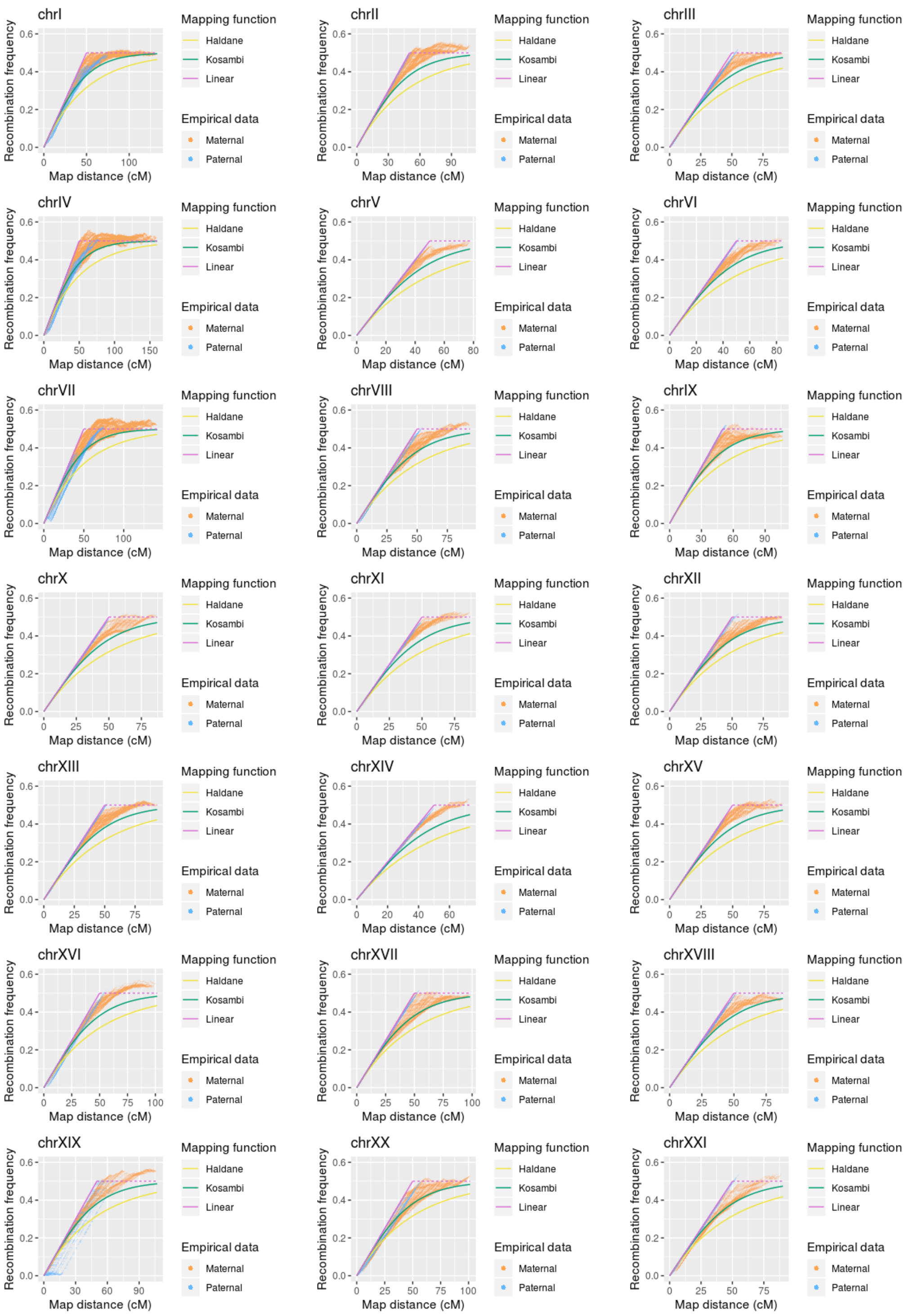

Figure S3. Empirical recombination frequencies in the 21 three-spined stickleback chromosomes. Solid lines show the three inverse mapping functions; the linear function was capped at 0.5, shown with the dashed line. Each orange and blue dot is a marker pair in maternal and paternal data, respectively.

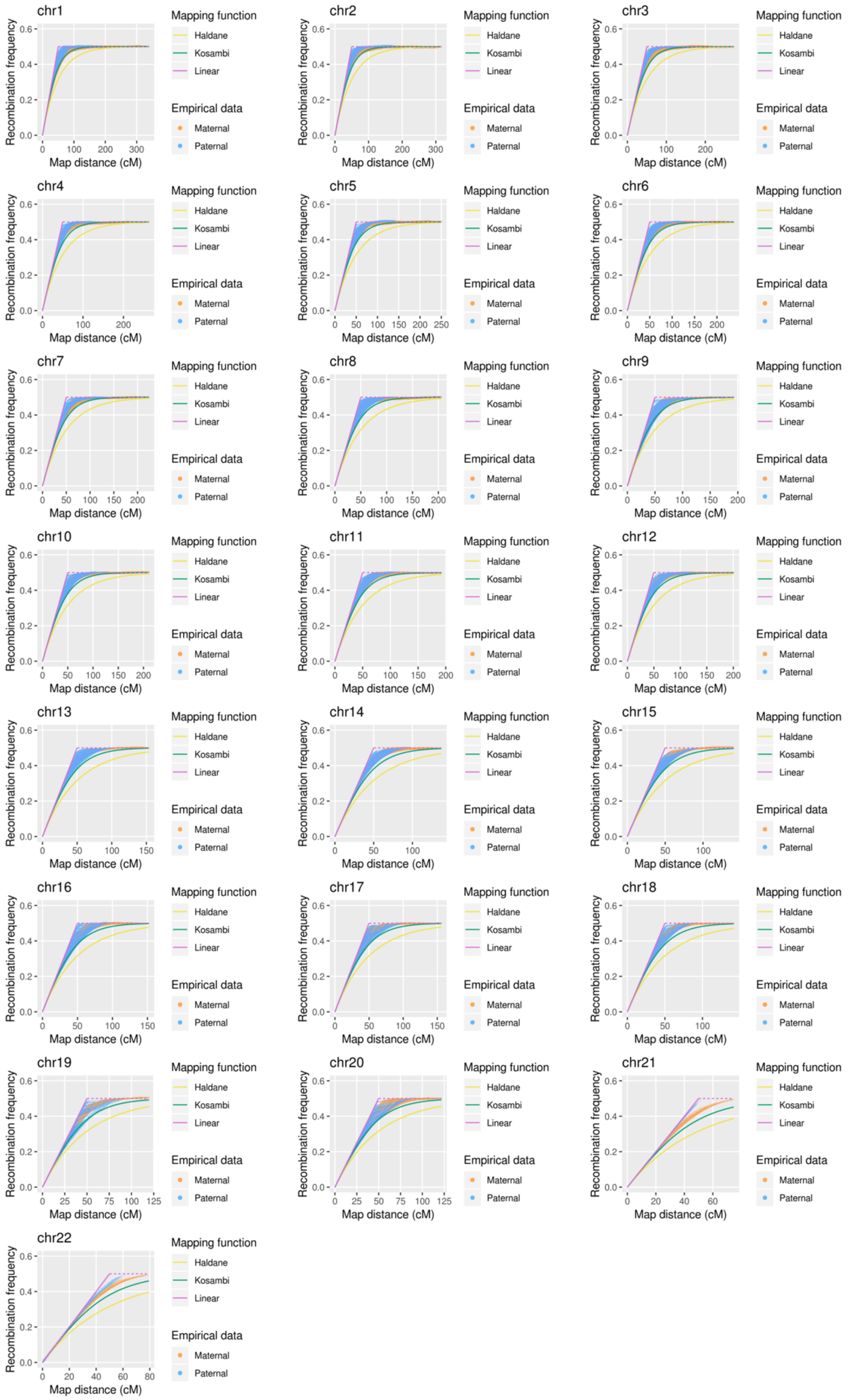

Figure S4. Empirical recombination frequencies in the 22 human autosomes. Solid lines show the three inverse mapping functions; the linear function was capped at 0.5, shown with the dashed line. Each orange and blue dot is a marker pair in maternal and paternal data, respectively.

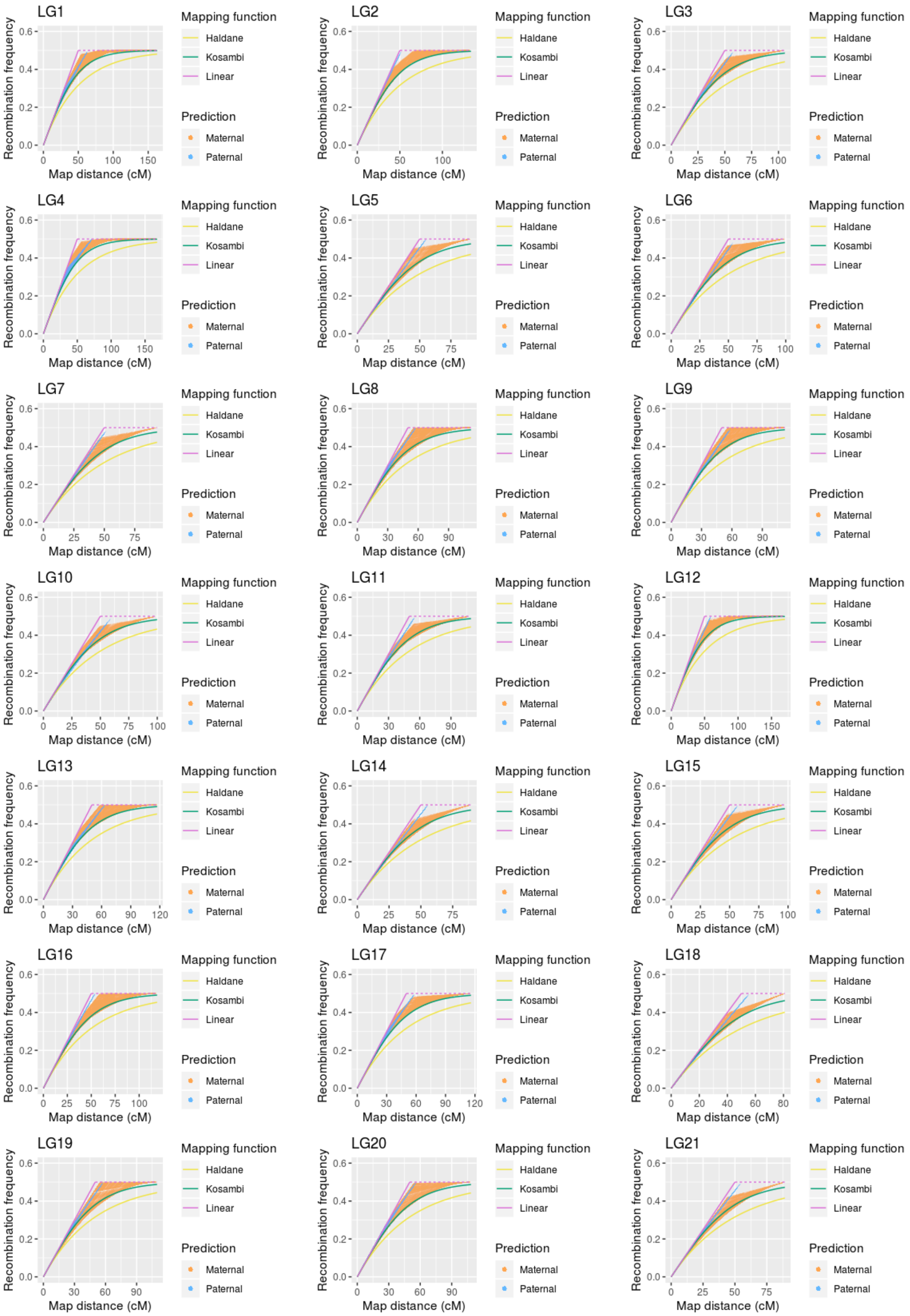

Figure S5. Recombination frequencies predicted with the $p_0(k)$ function for the 21 nine-spined stickleback chromosomes.

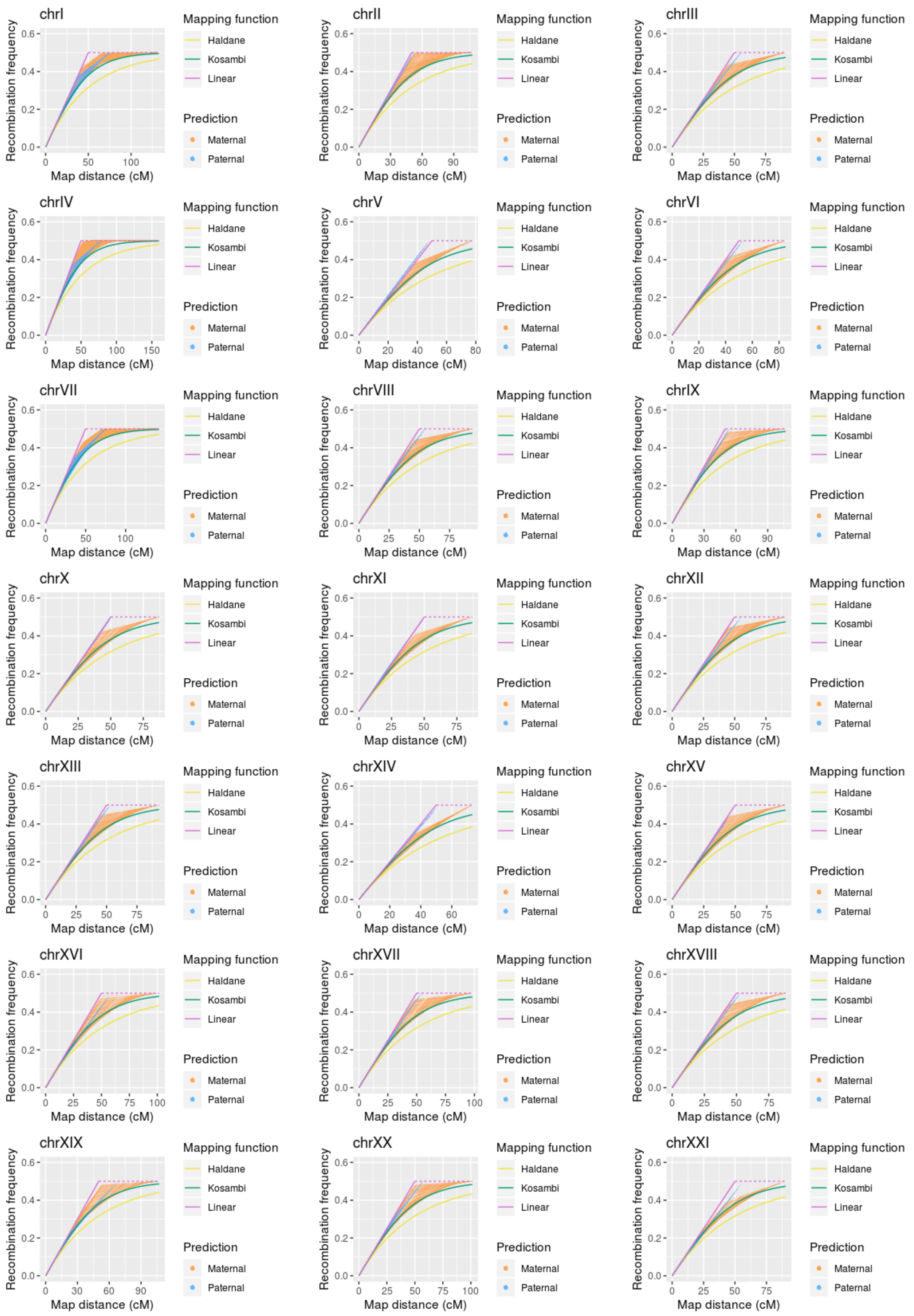

Figure S6. Recombination frequencies predicted with the $p_0(k)$ function for the 21 three-spined stickleback chromosomes.

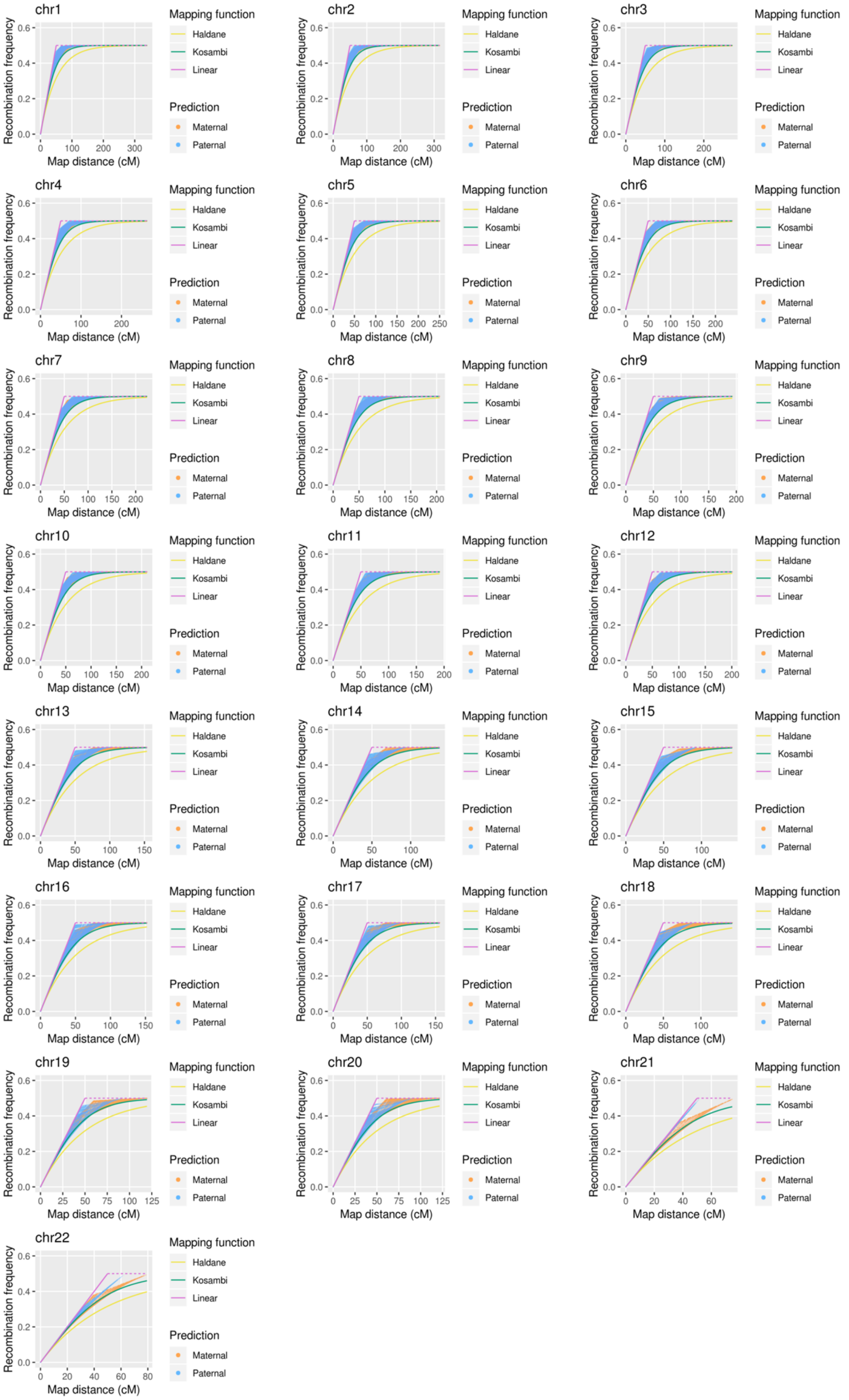

Figure S7. Recombination frequencies predicted with the $p_0(k)$ function for the 22 human autosomes.

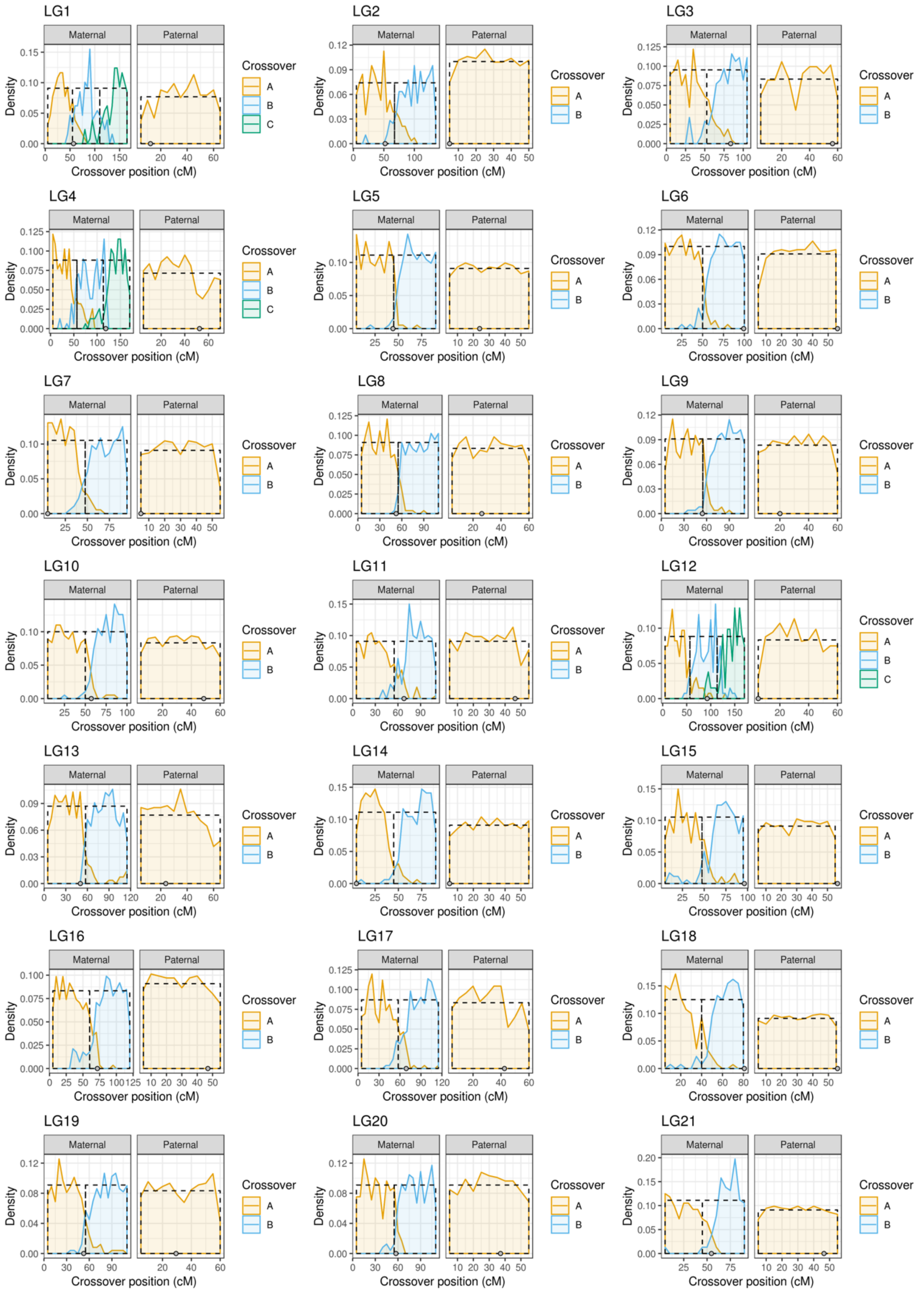

Figure S8. Empirical distributions of gametic crossovers per map distance in the 21 nine-spined stickleback chromosomes. Distributions are calculated from gametes whose crossover count is equal to the mode of the bivalent crossover count. Letters A–C refer to the order of the crossovers. Dashed rectangles show the assumed distributions of the crossovers as implemented in the $p_0(k)$ function. Centromeres are shown with the grey dots.

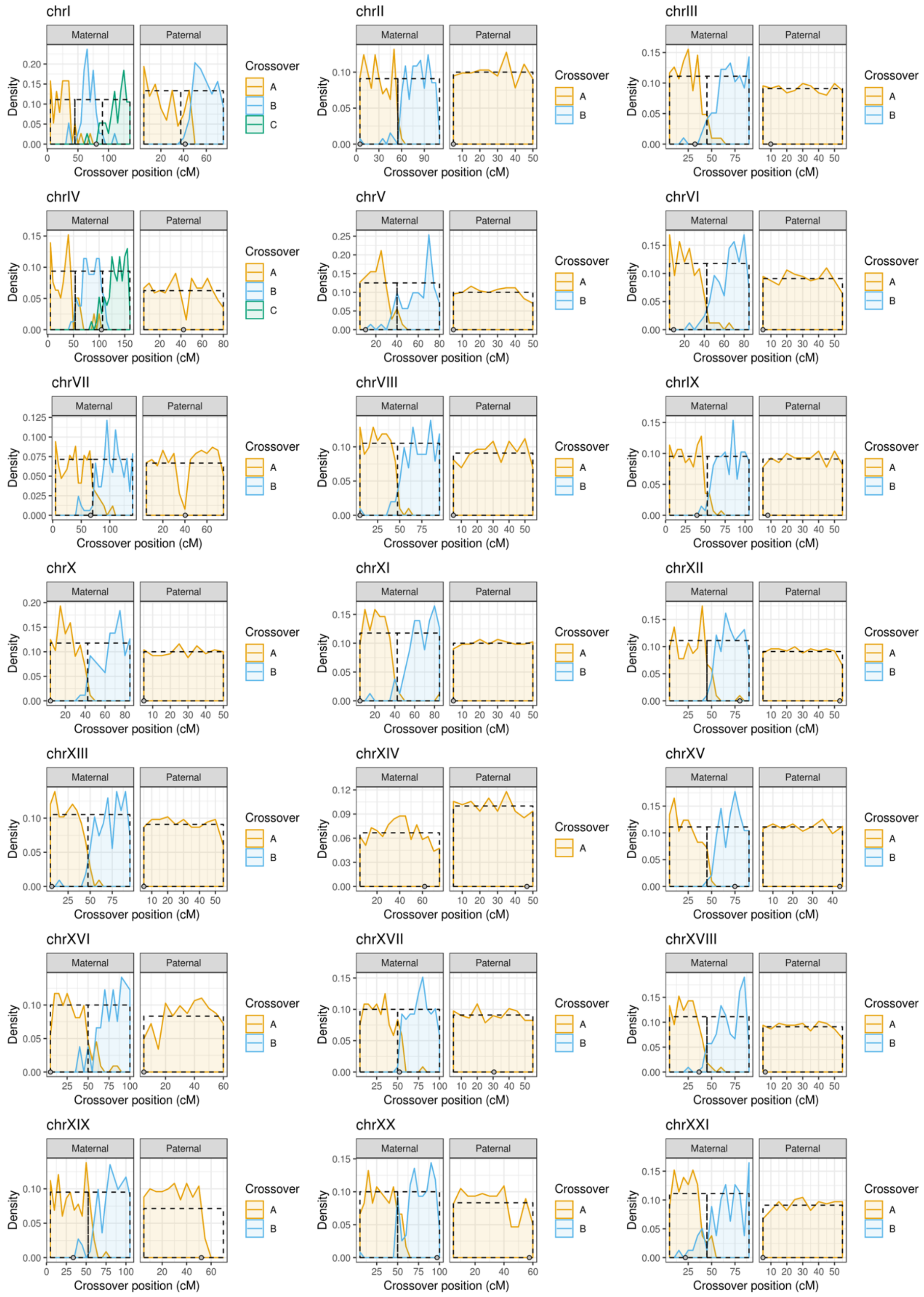

Figure S9. Empirical distributions of gametic crossovers per map distance in the 21 three-spined stickleback chromosomes. Distributions are calculated from gametes whose crossover count is equal to the mode of the bivalent crossover count. Letters A–C refer to the order of the crossovers. Dashed rectangles show the assumed distributions of the crossovers as implemented in the $p_0(k)$ function. Centromeres are shown with the grey dots.

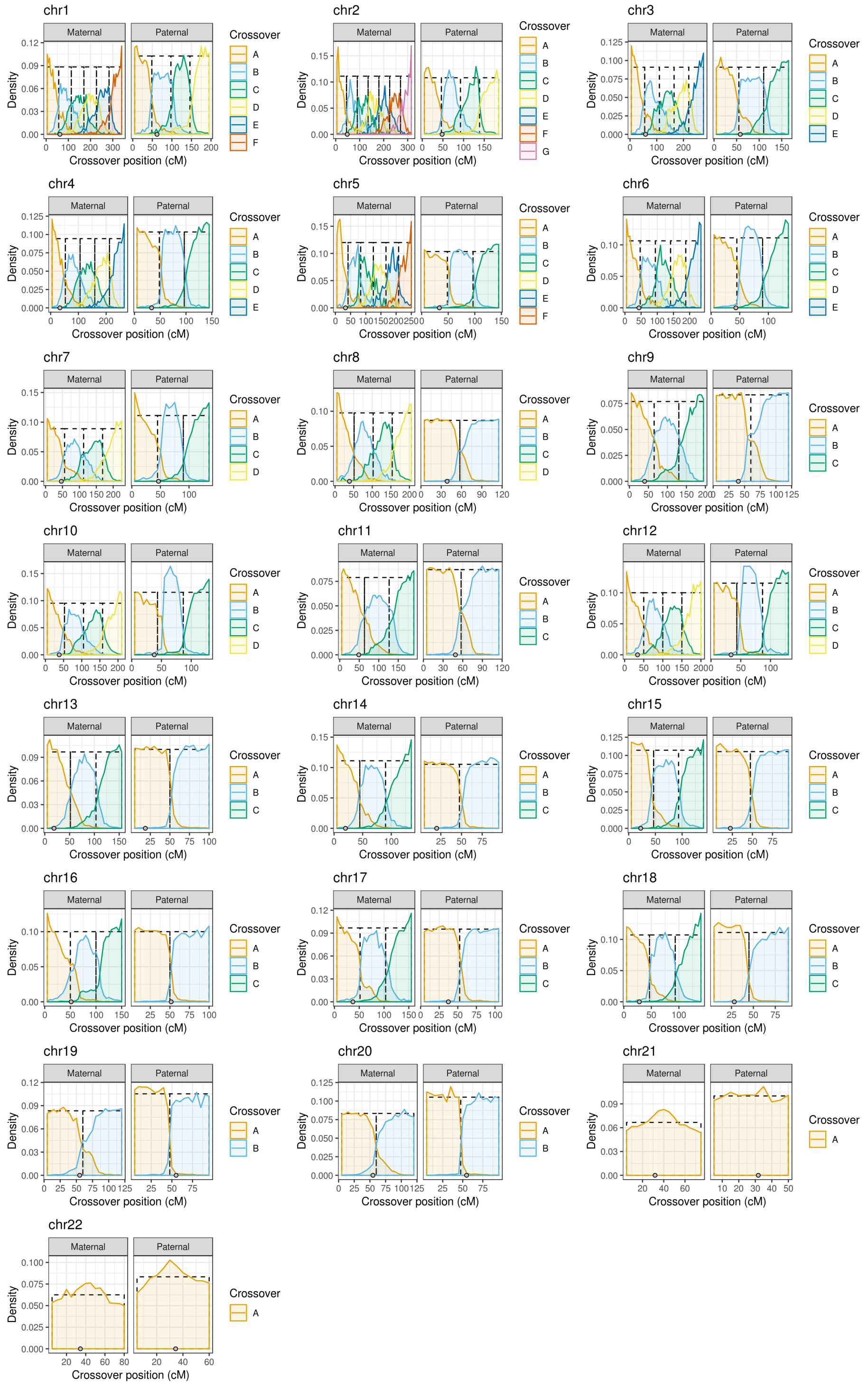

Figure S10. Empirical distributions of gametic crossovers per map distance in the 22 human chromosomes. Distributions are calculated from gametes whose crossover count is equal to the mode of the bivalent crossover count. Letters A–G refer to the order of the crossovers. Dashed rectangles show the assumed distributions of the crossovers as implemented in the $p_0(k)$ function. Centromeres are shown with the grey dots.

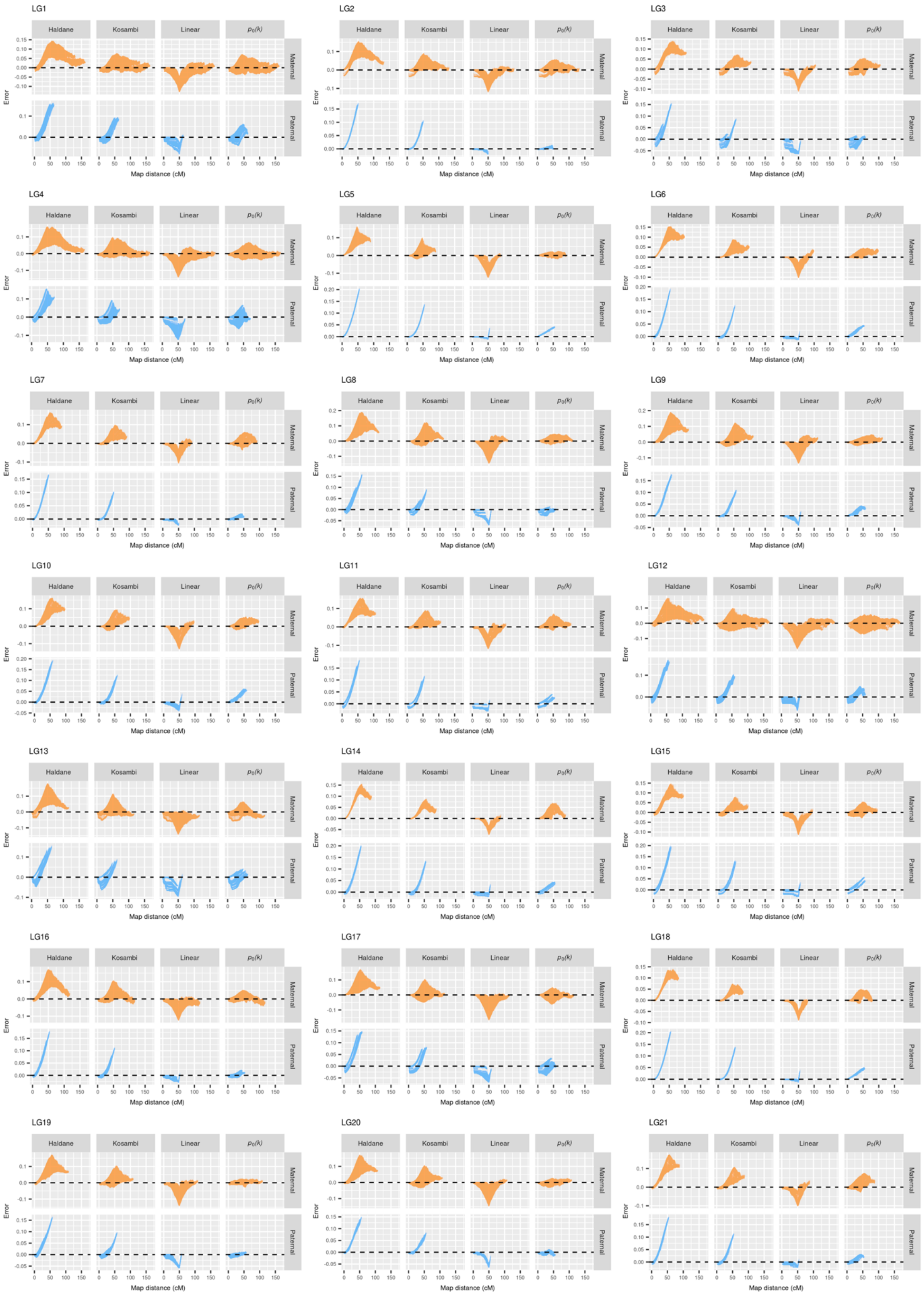

Figure S11. Error, i.e. the differences between empirical and predicted recombination frequencies with the different functions for the 21 nine-spined stickleback chromosomes.

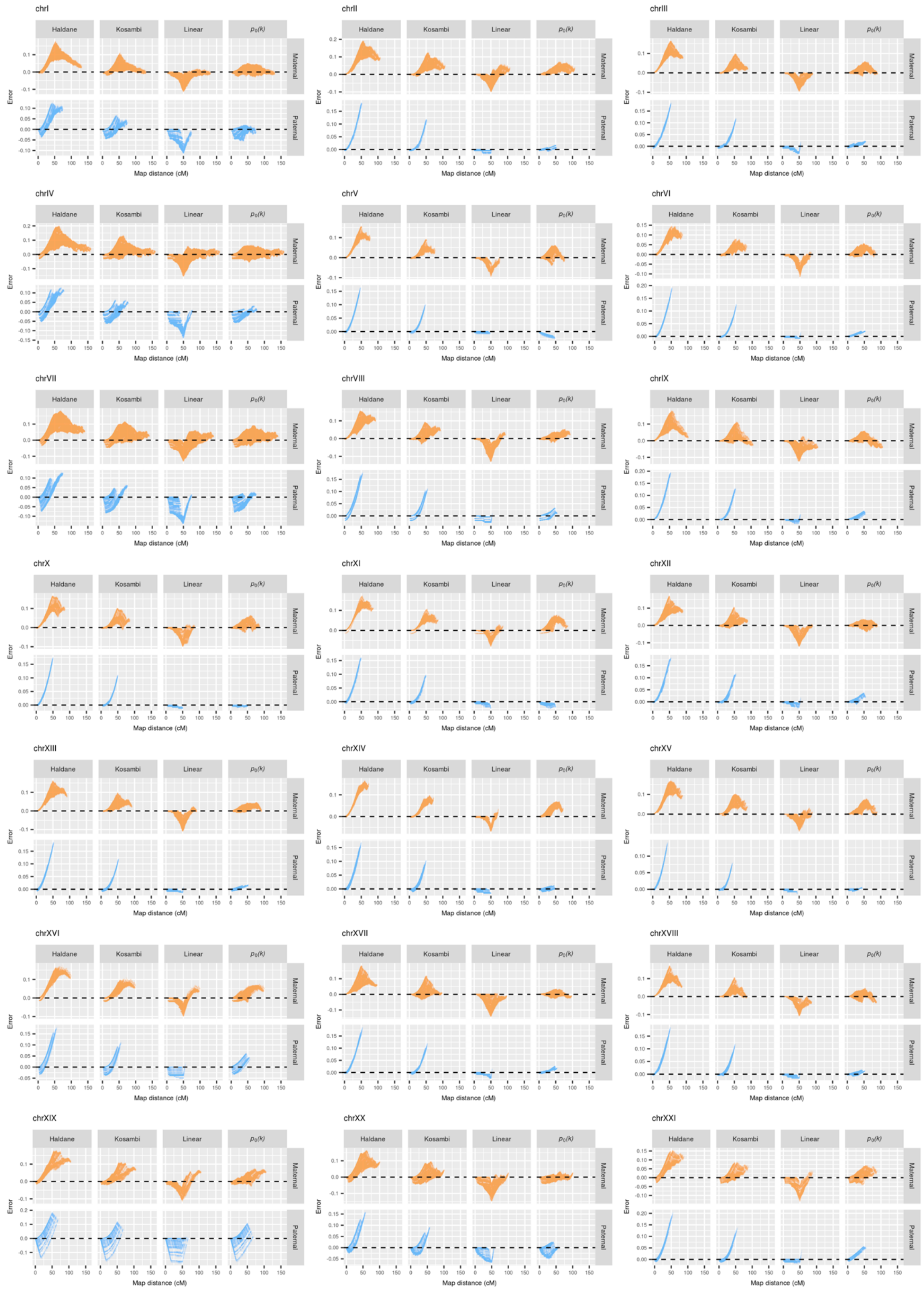

Figure S12. Error, i.e. the differences between empirical and predicted recombination frequencies with the different functions for the 21 three-spined stickleback chromosomes.

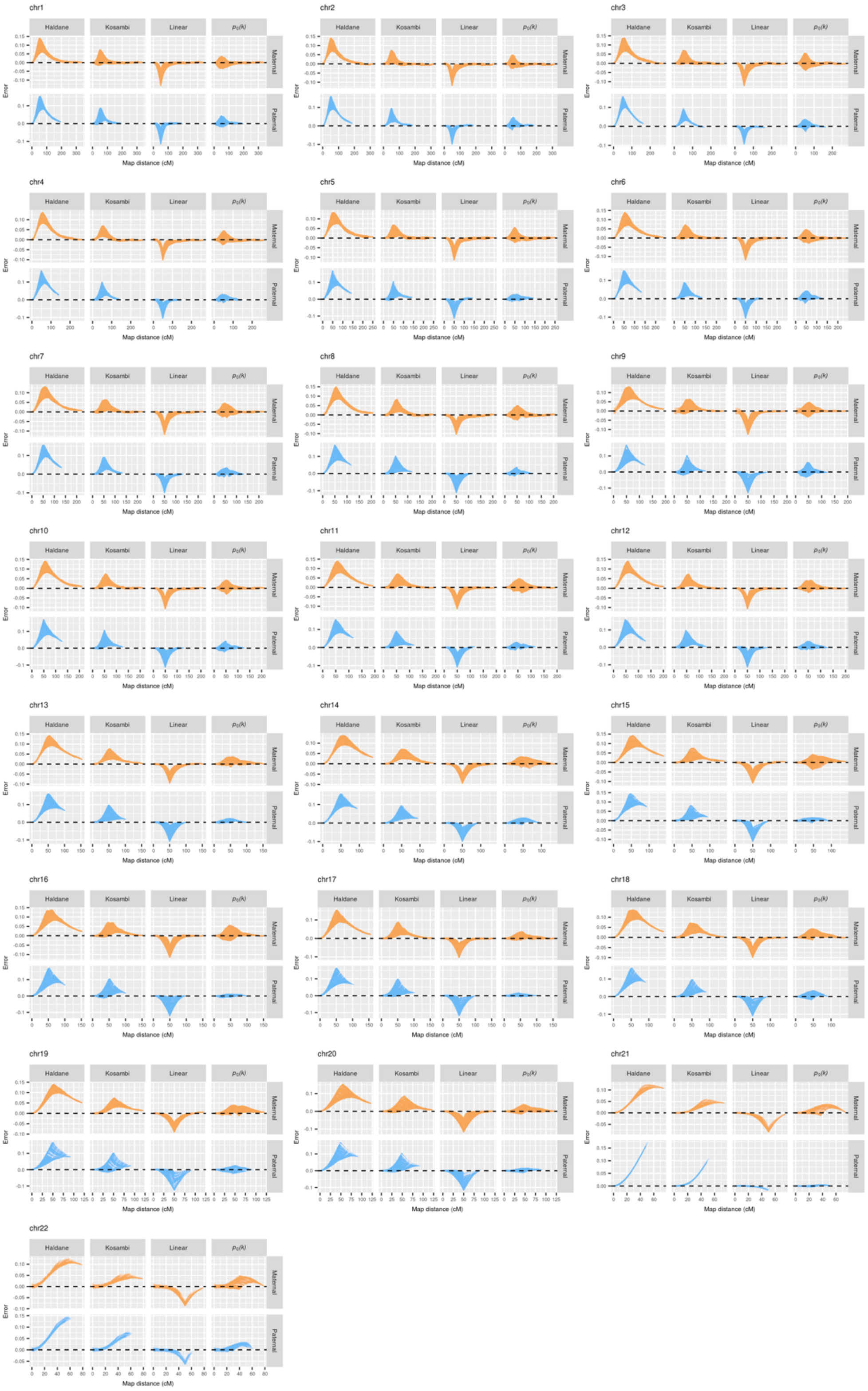

Figure S13. Error, i.e. the differences between empirical and predicted recombination frequencies with the different functions for the 22 human chromosomes.
